# A Neuropsychiatric View of Insomnia

**Published:** 1970-04

**Authors:** P. K. G. Warren

**Affiliations:** Consultant Psychiatrist, Barrow Hospital, Barrow Gurney, Near Bristol


### A Neuropsychiatric View of Insomnia by

**P. K. G. Warren, M.B., B.S., M.R.C.P., D.P.M.**

Consultant Psychiatrist, Barrow Hospital,Barrow Gurney, Near Bristol.

As Nathaniel Kleitman (1963) remarked in what remains a classic study of sleep and wakefulness, there are more books and articles devoted entirely, or mainly, to the discussion of insomnia than to any other trouble connected with sleep, but they are for the most part too general to comment upon.

Goethe wrote:
"I should be glad if, when people come to a clear understanding in natural science, they would stick to the truth, and not go transcendent again after all has been done in the region of the comprehensible". (Conversations).

A great deal of work has been done on sleep in recent years, in centres all over the world, on man and on animals, and from the standpoint of different disciplines. I shall try to summarise some of this work which seems to me interesting and relevant to the subject of insomnia.

Changes in the electroencephalogram in relation to behaviour and level of awareness have probably been most clearly described by Lindsley (1952) (Table I).

**TABLE I. Psychological States and Their EEG, Conscious and Behavioural Correlates**

| Behavioural Continuum | Electroencephalogram | State of Awareness | Behavioural Efficiency |
|---|---|---|---|
| Strong, excited emotion; fear, rage, anxiety. | Desynchronized: low to moderate amplitude; fast mixed frequencies | Restricted awareness: divided attention; diffuse, hazy: 'confusion' | Poor: lack of control, freezing up, disorganised |
| Alert attentiveness | Partially synchronized; mainly fast low-amplitude waves | Selective attention, but may vary or shift; 'concentration' anticipation; 'set' | Good; efficient, selective, quick reactions; organized for serial responses |
| Relaxed wakefulness | Synchronized: optimal alpha rhythm | Attention wanders — not forced; favour free association | Good: routine reactions and creative thought |
| Drowsiness | Reduced alpha and occasional low-amplitude slow waves | Borderline partial awareness; imagery and reverie; "dream-like" states | Poor: unco-ordinated, sporadic, lacking sequential timing |
| Light sleep | Spindle bursts and slow waves (larger); loss of alphas | Markedly reduced consciousnes (loss of consciousness); dream state | Absent |
| Deep sleep | Large and very slow waves (synchrony but on slow time bases); random irregular pattern | Complete loss of awareness (no memory for stimulation or for dreams) | Absent |
| Coma | Isoelectric to irregular large slow waves | Complete loss of consciousness; little or no response to stimulation; amnesia | Absent |
| Death | Isoelectric: gradual and permanent disappearance of all electrical activity | Complete loss of awareness as death ensues | Absent |

This description suggests that there are three main kinds of natural sleep and further studies have defined a fourth, and most interesting stage, known as paradoxical or rapid eye movement (REM) sleep, which is found in both man and animals, and first described by Dement and Kleitman (1957). Detailed all-night studies of these stages have since been made by many workers (Jouvet, 1965; Oswald, 1966, Luce and Segal, 1969; Evans and Jones, 1969) and a number of pertinent observations made:

The night consists normally of an initial period of deep slow-wave sleep, punctuated by 4 or 5 REM periods, occurring with a periodicity of about 90 minutes. Deep sleep is not remembered, though some mental life occurs in it, as occasional dream reports have been obtained when people were aroused from this stage. Sleep walking and nocturnal enuresis are initiated, and most muscular movements occur in this stage.

Dream reports are obtained almost universally on arousal from paradoxical (REM) sleep and the dreams often remembered. This stage is called paradoxical because an EEG pattern of rapid cortical activity is coupled with a behavioural stage of deep sleep—if the latter is judged by almost total muscular atonicity and, in fact, a considerable raising of the waking threshold (Jouvet, 1967). It is thought that the rapid eye movements represent following of visual images in dreams. Paradoxical sleep is the first sleep in narcolepsy and is the stage in which "sleep paralysis" and nightmares occur. Amphetamine and monoaminoxidase inhibitors increase the proportion of REM sleep; barbiturates greatly reduce it, but barbiturate withdrawal results in an increase of REM sleep, dreaming and nightmares for a period as long as a month. Sleep laboratory studies of people who report little or no sleep usually show a normal pattern; the paradoxical phases are remembered and equated with waking, whereas long periods of deep sleep are forgotten and, of course, the sense of time is totally disorganised in all phases of sleep.

The pattern of sleeping and waking depends on a complex relationship between neural and chemical elements: There are small collections of cells in the brain-stem, pons and in the region of the third ventricle, forming part of the reticular system, stimulation of some of which will cause arousal and others, sleep. The latter cells have a high serotonin content and, in cats, 80% destruction of these cells resulted in almost complete insomnia (Jouvet, 1967). Instillation of serotonin in the brain-stem caused cats to drop immediately to sleep in their food-bowls (Koella et al., 1965). Jouvet (quoted in Luce and Segal, 1969) abolished REM sleep in cats for 4-5 days with a single injection of reserpine, and reversed this effect with DOPA, a precursor of noradrenaline. Experimentally, dialysed brain-blood from a sleeping animal has produced sleep in a waking animal (Monnier and Hosli, 1964). Clearly the cortex takes part in the sleeping pattern, for not only is sleep in part a learned response and a habit, but it is common experience, which has been confirmed by experimental observation (Oswald, 1966), that significant stimuli cause arousal, whereas equally intense stimuli, which are not significant for the particular individual, do not. Thus the mother wakes at the first cry from her child, while the trains go by unheeded and father snores throughout.

There is a diurnal periodicity in a wide range of physiological functions as well as in sleeping and waking (Mills, 1969), and these variations, known as Circadian Rhythms, are found in the simplest of organisms, as well as in man (Bunning, 1967). When men are put in conditions isolated from the usual means of telling the time, such as in caves, North of the Arctic Circle (Oswald, 1966) or in conditions of experimental sensory deprivation, it is found that they live on a day slightly longer than 24 hours, and that about 8 hours' sleep occurs in that period. Numerous medical and psychiatric conditions which disturb Circadian Rhythms (Knapp, 1969) also disturb sleep, as does jet travel across the meridians of time; with regard to the latter, adaptation occurs in about four days. Shift work, although it has its problems, gives rise to many complaints, and has a very variable effect on individuals (Taylor, 1969), causes far less disturbance of the sleep pattern than one might suppose. A controlled study showed that shift workers took more sleep than controls, had fewer disturbances of the main sleep period, and that they built up a sleep debt while working, which was paid off by long naps during days off.

That we spend about a third of our lives sleeping and that we need this is beyond doubt. Animals continuously deprived of sleep eventually die; humans experimentally deprived of sleep developed impaired performance and transient psychotic states, and sleep-deprivation is a well known interrogator's tool in the process of brain-washing and inducing conversion and false confessions (Sargant, 1959). Prolonged sleep loss produces a marked reduction of alpha activity in the EEG, which overrides circadian variations and individual differences (Naitch et al, 1969). There is evidence, too, that we need both deep and paradoxical, dreaming sleep, for selective deprivation of either leads, as the case may be, to an increased proportion of deep or REM sleep in the ensuing undisturbed nights. This has led people to suggest that we need to dream, which is intriguing, but for which as yet there is no conclusive evidence. Freud (1929) likened the dream to a night watchman, a guardian of sleep, whose purpose it is to protect sleep from interruption. If the censorship, as he termed it, feels powerless against some dream wish which threatens to overthrow it, it then, instead of making use of distortion, destroys sleep by bringing about an access of anxiety. Because of partial abrogation of the censorship in favour of sleep at night, forbidden wishes can become active and he goes on to say, "There are nervous people suffering from insomnia who confess that their sleeplessness was voluntary in the first instance; for they did not dare to go to sleep because they were afraid of their dreams." Except to adduce evidence that we need sleep, we are still not in a position to say exactly why we have this need. Evans (1969) drew an analogy with the computer, which has to be programmed when it is "off-line", and suggested that sleep might be an "off-line" period when the brain processed data acquired during the day, related it to similar and past experience, and built up the memory store.

People vary a great deal in the amount of sleep they seem to need for optimum efficiency, but the average is the traditional 8 hours. Sleep habits, in so far as they are learned, are probably acquired in childhood. The difference in individual patterns can give rise to difficulties, for example in marriage:

"She has to go to bed at ten. I do at midnight. Then when I go to bed, she's already asleep."

"She likes a lot of blankets. I don't. So we had to take twin beds and that ruined our sex life".

Although we all build up often quite elaborate rituals for going to sleep it is remarkable how these rituals have been observed to go in times of disaster, as with earthquakes, war-time experiences and concentration camps.

A decreasing amount of sleep is taken as age increases and this is paralleled by increasing complaints of insomnia with age. McGhie and Russell (1962) found that 7% of people under 40 said they had less than 5 hours sleep at night, and this rose to 22%, with frequent early waking in the over-70's. There is also a sex difference, for 30% of females over 65 took longer than 90 minutes to fall asleep, but few of the men. Dissatisfaction with sleep, as reflected by the demand for drugs, increases with age, so that by the age of 75, 45% of women were regularly taking sleeping pills. In 1962, 10% of all general practice N.H.S. prescriptions were for sleeping pills.

Insomnia is commonly described according to the time at which it occurs, i.e. difficulty in getting off to sleep, frequent waking with nightmares, or early waking, but for the purpose of this discussion I find Hemphill's (1957) classification of the causes of insomnia useful:

(a) Over-excitement and anticipation
(b) Anxiety and psychoneurosis
(c) Psychotic conditions, such as mania and depression
(d) Disturbed sleep pattern in senility
(e) The loss of the sleep habit
(f) Pain and physical illness.

(a) In **over-excitement and anticipation,** as before a wedding or an examination, the patient may get little or no sleep without a hypnotic. A good dose of a quick-acting barbiturate is probably best, but should be given not less than 8 hours before rising so as to avoid hang-over. Hypnotics may be important also in the treatment of insomnia due to recent events which have caused anxiety, or after bereavement. But here I think we might pause to reflect that the first prescription of a barbiturate may be a serious event in a person's life. I have observed that many people may take barbiturates for years and never exceed the prescribed dose, but for reasons already given, few find it easy to stop them. A few demand excessive doses, and barbiturates remain a favourite resort of the suicide. Only too often, patients are started on barbiturates in hospital by an almost automatic prescription, perhaps to ensure that the overworked houseman gets a night's sleep rather than the patient! If a hypnotic drug has to be used we can, these days, often find a non-barbiturate, and most of us have our favourites, which we have learned to use. Most non-barbiturates however, may also give rise to dependence, though less readily, and can cause death with gross overdosage. At the risk of advertising, I should like to put in a good word for nitrazepam. With a large number of anxious mental hospital in-patients, I have had no more complaints from patients or staff with nitrazepam than with barbiturates. A recent controlled study (Matthew et al., 1969) has shown it to be equally as effective as butobarbitone and a search of the literature and their own experience produced no evidence of dependence, withdrawal symptoms or death from overdose with as many as 80 tablets. If it seems that only a barbiturate will do, then I think Hemphill (1957) was wise to advise that after the patient has had two or three nights' sound sleep he should be instructed to do without drugs, even at the expense of a restless night, and thereafter not to take sleeping pills on more than three nights in a week.

(b) **Anxiety and psychoneurosis** are by far the most common and important causes of chronic insomnia. Adler (1929) described nervous insomnia in terms similar to one of Eric Berne's (1968) destructive psychological games, where the symptom is exaggerated in importance so as to serve as a signal and weapon against spouse, doctor or employer, and as evidence of illness and an excuse for the avoidance of difficulties. The patient may demand ten hours' sleep at the cost of a heavy hangover in the morning; this in itself can be responsible for inefficiency and inability to cope with work or house, which may have more serious consequences than the original circumstances and do much to make the neurotic condition chronic. The treatment is primarily directed to the neurosis rather than the insomnia but, in some cases, the vicious circle of tension and drug dependence may be broken by a period of continuous narcosis with non-barbiturate drugs in hospital (Bradfield, 1969).

(c) In **psychotic conditions,** a change in the sleep pattern may be an early sign and indication for specific treatment of the illness.

(d) The **disturbed sleep pattern in senility** is a constant problem to the patient and to those who attend him, and there are often multiple causes which require attention; physical causes, such as cardiac or respiratory disease, or a full bladder, organic brain disease which, for various reasons, is particularly liable to give trouble at night, and morbid depression. Barbiturates often aggravate restlessness due to organic brain disease, and a combination of a suitable tranquilliser by day and a non-barbiturate at night is far better. It is important in these cases to try to establish a regular routine for going to bed, getting up, attention to bladder and bowels, and for meals and recreation.

(e) **Loss of the sleep habit** disturbs tense and anxious people more than others who are basically more sanguine and phlegmatic, and is liable to happen with mothers who have been kept awake by babies, to shift workers and to those who have been kept alert nursing a sick person. Because the cause may continue and because of the personality factor, these cases are difficult to treat. One must try by various means to allay the anxiety, to break the chain of sleeplessness by the use of hypnotics in the manner suggested earlier, and to try to establish a more normal routine.

(f) The management of insomnia due to **pain and physical illness** is a subject in itself and, as I have been taking a mainly psychiatric view of insomnia, I shall only say that it is of course bound up with the treatment of the particular physical condition, but that one broken leg or case of heart failure is, for psychiatric reasons, not the same as another, and that the emotional reactions and attitudes, even in the most obvious physical case, should never be forgotten.

### REFERENCES

**Adler, A.** (1929): "The Practice and Theory of Individual Psychology", Kegan Paul, Trench, Trubner and Co., London.

**Berne, E.** (1968): "Games People Play", Penguin Books, London.

**Bradfield, A. M.** (1969): Personal communication.

**Bunning, E.** (1967): "The Physiological Clock", 2nd Ed., Springer-Verlag, Berlin.

**Dement, W., and Kleitman, N.** (1957): "Cyclic Variations in the E.E.G. during sleep and their relation to Eye Movements, Bodily Motility and Dreaming", Electroenceph. Clin. Neurophysiol. **9,** 673-690.

**Evans, C., and Jones, B.** (1969) in "Sunday Times Magazine", 16.2.69.

**Freud, S.** (1929): "Introductory Lectures on Psychoanalysis", George Allen and Unwin Ltd., London.

**Hemphill, R. E.** (1957): "The Use and Misuse of Sedative and Hypnotic Drugs", Med. J. South-West **72,** 12-22.

**Jouvet, M.** (1965): "Paradoxical Sleep—A Study of its Nature and Mechanisms", in "Sleep Mechanisms," ed. Akert, K., Bally, C., and Schade, J. P., Elsevier, Amsterdam.

**Jouvet, M.** (1967): "The Sleeping Brain", Sci. J., **3,** 105-110.

**Kleitman, N.** (1963): "Sleep and Wakefulness', Univ. of Chicago Press.

**Knapp, M. S.** (1969): "Nocturia and Reversed Circadian Rhythms", J. roy. Coll. Phycns. London 3, 398-405.

**Koella, W. P., Trunca, C. M., and Czicman, J. S.** (1965): "Serotonin: Effect on Recruiting Responses of the Cat", Life Sci. **4,** 173-181.

**Lindsley, D. B.** (1952): "Psychological Phenomena and the E.E.G.", Electroenceph. Clin. Neurophysiol. 4, 443-456.

**Luce, C. G., and Segal, J.** (1969): "Sleep and Dreams", Panther Science, London.

**McGhie, A., and Russell, S. M.** (1962): "The Subjective Assessment of Normal Sleep Patterns", J. ment. Sci. **108,** 642-654.

**Matthew, H., Proudfoot, A. T., Aitken, R. C. B., Raeburn, J. A., and Wright, N.** (1969): "Nitrazepam — a Safe Hypnotic", Brit. med. J. **3,** 23-25.

**Mills, J. N.** (1969): "Man Underground", J. roy. Coll. Phycns. Lond. **3,** 329-333.

**Monnier, M., and Hosli, L.** (1964): "Dialysis of Sleep and Waking Factors in the Blood of the Rabbit", Science **146,** 796-797.

**Naitch, P., Kales, A., Kollar, E. J., Smith, J. C., and Jacobson, A.** (1969): "Electroencephalographic Activity after Prolonged Sleep Loss", Electroenceph. Clin. Neurophysiol **27,** 2-11.

**Oswald, I.** (1966): "Sleep", Penguin Books, London.

**Sargant, W.** (1959): "Battle for the Mind", Pan Books, London.

**Taylor, P. J.** (1969): "The Problems of Shift Work", J. roy. Coll. Phycns. Lond. **3,** 370-383.

